# A270 THE PREBIOTIC, INULIN, IMPACTS TUMORIGENESIS PROMOTION BY COLIBACTIN-PRODUCING

**DOI:** 10.1093/jcag/gwac036.270

**Published:** 2023-03-07

**Authors:** M Oliero, R Hajjar, T Cuisiniere, G Fragoso, A Calvé, M M Santos

**Affiliations:** 1 Nutrition and Microbiome Laboratory - Institut du cancer de Montréal - Centre de recherche du Centre hospitalier de l’Université de Montréal (CRCHUM); 2 Department of Medicine, Faculty of Medicine - Université de Montréal, Montreal, Canada

## Abstract

**Background:**

The prebiotic inulin has previously shown both protective and tumor-promoting effects in colorectal cancer. These discrepancies may be due to polyketide synthase-positive (pks+) Escherichia coli promoting carcinogenesis through the production of colibactin, a genotoxin that induces double-strand DNA breaks (DSBs).

**Purpose:**

In this study we investigated the impact of inulin on the genotoxicity of colibactin-producing bacteria.

**Method:**

E. coli strains Nissle (EcN), and NC101 (EcNC101) were grown in medium supplemented with inulin. Colibactin expression was assessed by luciferase reporter gene expression and Caco2 cells were used to assess colibactin-induced genotoxicity by γ-H2AX immunofluorescence analysis. ApcMin/+ mice received 2% dextran sodium sulfate (DSS) followed by oral gavage with EcNC101 and were fed a diet supplemented with 10% cellulose (control diet) or 10% inulin for four weeks.

**Result(s):**

Inulin enhanced EcNC101-induced expression of colibactin and DSB levels in Caco2 cells. Inulin supplementation in ApcMin/+ mice led to enhanced EcNC101 colonization and tumor progression.

**Conclusion(s):**

The presence of colibactin producing E. coli in the gut influences the outcome of inulin supplementation in CRC progression. Further studies are needed to investigate the interaction between dietary supplements and cancer-promoting bacteria.

**Please acknowledge all funding agencies by checking the applicable boxes below:**

CIHR, Other

**Please indicate your source of funding;:**

NSERC, Institut du cancer de montréal

**Disclosure of Interest:**

None Declared

